# Practical Sanitary and Economic Cooking

**Published:** 1890-09

**Authors:** 


PRACTICAL SANITARY AND ECONOMIC COOKING, adapted to persons of moderate and small means. By Mrs. Mary Hinman Abel. The Lomb Prize Essay. Published by the American Public Health Association.

This work deserves to be known and recommended by all physicians to patients who are so situated that economy in cooking, and a knowledge of how to get the most nutritive value from food, at the least cost, are necessary. The object of the little book is "purely an effort to better the condition of the home, and to make happier the family circle." A laudable enterprise. We have always claimed that a large amount of drunkenness is caused by the fact that the laboring poor, through ignorance, do not get the full nutritive value of the food they use; mostly through improper preparation. Hence, weakness; then a desire for stimulants; then drunkenness; finally an unhappy home. Make this book known to such, and it will do a work that all the temperance and prohibition societies have failed to accomplish. G. H. C.

